# The energy expenditure of people with spinal cord injury whilst walking compared to an able-bodied population

**DOI:** 10.4102/sajp.v72i1.255

**Published:** 2016-03-31

**Authors:** Jana Vosloo, M. Veronica Ntsiea, Piet Becker

**Affiliations:** 1 Department of Physiotherapy, University of the Witwatersrand, South Africa; 2 Biostatistics Unit, South African Medical Research Council, South Africa

## Abstract

**Background:**

In the field of spinal cord injury (SCI) research there is an emphasis on the ability to ambulate.

**Purpose:**

To determine the ambulation energy expenditure (EE) and factors that affect ambulation EE in SCI participants compared to able-bodied participants.

**Methods:**

This was a cross-sectional study. Participants were recruited from seven SCI rehabilitation units within the Johannesburg area. The following were used: demographic questionnaire to capture participants’ characteristics, modified Ashworth scale for spasticity; goniometer for range of movement (ROM); American Spinal Injury Association (ASIA) scale for patient classification; accelerometer for EE and the six-minute walk test (6MWT) for endurance. Characteristics of the study participants were summarised using descriptive statistics. Data were analysed as follows: two-sample *t*-test for comparison between the able-bodied and SCI sample and Pearson product moment correlations for relationship between identified factors and EE.

**Results:**

Participants comprised 45 in the SCI group and 21 in the able-bodied group. The mean energy expenditure per metre (EE/m) for the SCI participants was 0.33 (± 0.29) calories compared to 0.08 (± 0.02) calories for the able-bodied participants. A decrease in walking velocity resulted in an increase in EE. For SCI participants, every decrease in degree of hip flexion ROM resulted in a 0.003 increase in EE/m walked. A unit decrease in velocity resulted in an increase of 0.41 in EE/m walked. Energy expenditure per metre decreased from ASIA A to ASIA D. Crutch walking utilised 0.34 calories per metre less energy than walking frames (*p* = 0.03).

**Conclusion:**

Based on this study’s findings, factors to consider in order to maximise energy efficiency whilst walking are maintaining hip flexion ROM and optimising velocity of walking.

## Introduction

In the field of spinal cord injury (SCI) research there is an emphasis on ability to ambulate as a functional outcome (Jackson *et al*. 2008). Walking for routine activities of daily living (ADLs) requires ability to cover more than 250 m and to spend on average at least 10 min walking (Ulkar *et al*. 2003).

In people with SCI, gait patterns are significantly altered because of decreased strength, endurance, proprioception and spasticity (Jackson *et al*. 2008). These impairments may also result in the use of orthotic devices which can increase energy demands (Field-Fote & Fluet 2001). When energy expenditure (EE) during orthotic-assisted gait is compared to able-bodied individuals, as expected, the energy cost is far higher during orthotic gait (Kawashima *et al*. 2006). Another factor that would impact walking efficiency is the level of neurological impairment. High levels of SCI lesions result in high physiological intensity when walking (Kawashima *et al*. 2006).

The excess EE for ambulation and the burden on particularly the upper limbs present a challenge in achieving functional walking. It is therefore vital to explore options to reduce the excess physiological load (Kawashima *et al*. 2006). The following factors have been found to affect ambulation for people with a variety of medical conditions, including SCI: being overweight (Yamakawa *et al*. 2004), increased age (Scivoletto *et al*. 2003), spasticity (Mahoney *et al*. 2007), pain (Richards *et al*. 2004), range of movement (ROM) (Kawashima *et al*. 2006), level of lesion (Kawashima *et al*. 2005), assistive devices (Kawashima *et al*. 2006), extent of treatment (Scivoletto *et al*. 2008) and duration since the injury (Kirshblum *et al*. 2004). Even though these factors were established using a variety of medical conditions, all of these factors are also applicable to patients with SCI. Expenditure of a considerable amount of metabolic energy is also required when walking (Kuo 2007).

There is a dearth of literature on the effect of the above-mentioned factors on EE whilst walking for people with SCI when compared to able-bodied people. The purpose of this study was to determine ambulation EE and also establish factors that affect ambulation EE in SCI participants compared to able-bodied participants. Having evidence of walking EE for individuals with SCI compared to an able-bodied population would enable therapists to draw conclusions regarding the discrepancies that may exist and quantify these discrepancies.

## Methods

This was a cross-sectional study using a questionnaire, direct observations and physical assessments. Ethical clearance was applied for and obtained from the Committee for Research on Human Subjects of the University of the Witwatersrand (clearance number: M110944). The required sample size was determined as follows: from the pilot study performed with 10 participants in each group, the able-bodied participants’ mean EE had a standard deviation of ± 0.018 calories per metre (cal/m). The standard deviation for the able- bodied participants was used because they were expected to have a relatively lower EE than SCI participants and thus the sample size requirements would be relatively higher than when using high scores for the SCI participants. Using this standard deviation, a sample size of at least 28 SCI participants and at least 14 able-bodied participants had 90% power to detect change in EE within a group. A standard deviation of 0.018 cal/m was used and testing was two-sided at the 0.05 level of significance.

Participants for the study were recruited from seven conveniently selected SCI rehabilitation units (five private and two government) within the Johannesburg area of South Africa. These facilities were contacted on a weekly basis to enquire about suitable participants. A process of consecutive sampling was used until the required number of participants was reached. Recruitment for the able-bodied group was carried out by requesting therapists and other staff members, family and friends meeting the criteria to participate. Participants with SCI had to meet the following eligibility criteria:

- was ambulatory prior to SCI (to exclude those whose ambulation limitations were not as a result of SCI)
- has a complete thoracic level 10 and below or incomplete lesions at any spinal level
- comprehend and follow instructions
- walk 10 m unassisted (with or without walking aids and orthotics).

Able-bodied participants were included if they were comparable in age, gender and body mass index (BMI) to participants with SCI. The researcher provided potential participants with information, clearly stating the aims of the study and what was expected if they chose to participate. Informed consent was sought from those who were willing to participate in the study.

### Instruments used during the data collection process

A questionnaire was developed which captured the following information about the SCI participants: gender, age, date of SCI, use of orthotics and walking aids for mobility, extent of mobility (whether they walk therapeutically, walk indoors, walk outdoors, walk at work and walk in the community), duration since SCI, inpatient and outpatient therapy, therapy hours per day and duration of rehabilitation hospital stay. Other items that could influence EE such as BMI, level of the lesion, presence of pain and extent of rehabilitation were also included. The matched able-bodied participants only filled in age, gender and mobility. Their height and weight were measured and used to calculate their BMI. The BMI and EE were also recorded on the questionnaire sheet. Content validity of this questionnaire was established by asking therapists who work in the SCI rehabilitation units to assist with identification of factors that could influence EE of SCI participants whilst walking. This was done by asking them to comment on the questionnaire and via individual interviews.

Each SCI participant had the American Spinal Injury Association (ASIA) impairment scale assessment to classify the severity of their SCI (ASIA 2002; Lammertse *et al*. 2007). The researcher assessed both motor and sensory scores with the ASIA impairment scale to establish the level of the spinal cord lesion. The ASIA classification (ASIA 2002) is as follows:

- ASIA A: complete injury where no sensory or motor function is preserved in sacral segments S4–S5
- ASIA B: incomplete injury where sensory but not motor function is preserved below the neurologic level and extends through sacral segments S4–S5
- ASIA C: incomplete injury where motor function is preserved below the neurological level, and most key muscles below the neurologic level have muscle grade less than three (active full-range movement against gravity)
- ASIA D: incomplete injury where motor function is preserved below the neurological level, and most key muscles below the neurologic level have muscle grade greater than or equal to three
- ASIA E: normal sensory and motor functions.

Height and weight measurements were done for all participants in order to calculate BMI. The height measurement was done in supine on the plinth with legs and trunk straightened and ankles in a neutral position (plantargrade). No shoes were worn during this measurement. Arms were positioned by the participant’s sides. A mark was made on the surface at the top of the head and between the bases of the heels with the ankles in neutral. The participant then moved aside to allow measurement of the two points to establish the height. Measurements were done using a measuring tape. Prior to weight measurement the SECA 813 scale was calibrated by using a 3 kg dumbbell weight before each weight measurement was performed. The weight measurement was done by stepping onto the scale, which was positioned within the parallel bars. The participants were allowed to use the bars to climb onto the scale and to steady themselves until balanced. If the participant was dependent on orthotics to maintain balance, the assistive devices were weighed afterwards and the weight subtracted from the weight of initial measurement. If accurate weight measurement was difficult because participants were unable to statically stand without external support, it was noted on the demographic questionnaire. Weight was measured to the nearest 0.1 kg using the SECA 813 digital scale (Van Cauwenberghe *et al*. 2011). A BMI range of 18.5 kg/m^2^ – 24.9 kg/m^2^ was considered normal (WHO 2000).

The amount of energy expended whilst performing the walking assessment was evaluated using an RT3 triaxial accelerometer (Hale *et al*. 2007; Hendrick *et al*. 2010). The RT3 provides data measured in ‘activity units’ and can be converted to estimate EE in total calories or metabolic equivalent (METS) (Hale *et al*. 2007). For the purposes of this study, the evaluation of the most discrete movements that would influence the EE over a set distance was important. Data were recorded in one-second intervals. The walking assessment was performed with participants walking at a comfortable self-selected speed along the well indicated 10 m line, which was marked at 2.5 m intervals.

The six-minute walk test (6MWT) was used to establish endurance (Stuart, Holland & New 2009). No numeric adjustments in scoring were made for the use of walking devices, orthotics or pharmaceutical anti-spasmodics, but the use of these products were recorded in the demographic questionnaire. A Polar heart rate monitor was used to measure baseline and end of walking pulse rate as an indicator of cardiac regulation and physiological cost of gait (Arazpour *et al*. 2012). The Modified Ashworth Scale (MAS) was used to measure the intensity of spasticity where applicable (Kakebeeke *et al*. 2002). For the muscle which primarily performs flexion at a joint, the limb was positioned in maximal flexion and moved into maximal extension over one second. The opposite occurred when a muscle of primary extension was tested. A goniometer was used to measure the ROM for the lower limbs (Eriks-Hoogland *et al*. 2011).

Categorical data were summarised as numbers and percentages. Continuous data were summarised as means and standard deviations. Data were analysed for comparison between the able-bodied and SCI sample using the two-sample *t*-test. Fisher’s exact and chi-squared tests were used to establish the relationship between categorical variables. Pearson’s product moment correlations were used to establish the relationship between identified factors and ambulatory EE. Scheffé comparison was used to analyse the differences in EE whilst walking with different assistive devices. Multivariate stepwise regressions were performed to establish factors which had an influence on ambulatory EE. The factors which had significant relationships with EE from a univariate analysis were included in the stepwise multivariable regression. Factors which did not remain significant were removed.

## Results

The data collection period was January to November 2012. The sample consisted of 45 SCI and 21 able-bodied participants. The mean age for the SCI and able-bodied participants was 38.9 (± 13.6) and 34.7 (± 9.7) years respectively. There were more men in both the SCI and able-bodied groups (80% and 76.2% respectively). The mean BMI was 23.9 (± 5.1) kg/m^2^ for the SCI and 23.8 (± 4.4) kg/m^2^ for the able-bodied participants. The differences between age, gender and BMI of both groups were not statistically significant. Twenty percent of the SCI participants had increased tone bilaterally and throughout ROM.

Of the participants, 46.6% (*n* = 21) had an injury above T10 which was incomplete and therefore still enabled them to walk. Thoracic spinal cord injuries, T10–T12, were observed in 26.7% (*n* = 12) of participants. Lumbar spine injuries, L1–L5, also represented 26.7% of the SCI group. With regard to ASIA classification scores (Table 1), the mean motor and sensory scores are highest for ASIA D and progressively decrease with each category so that scores are lowest for ASIA A.

**TABLE 1:** American Spinal Injury Association classification motor and sensory scores.

ASIA	A		B		C		D	
			
	*n*	%	*n*	%	*n*	%	*n*	%
Number of participants	6	13	1	2	12	27	26	58
Mean sensory score	147.3	-	175	-	179.5	-	183.8	-
Mean motor score	54.8	-	65	-	68.4	-	74.1	-

*n* = 45.

ASIA, American Spinal Injury Association.

Most of the participants (38%) reported less than 6 months since SCI, 51.1% received outpatient therapy after discharge from an inpatient facility, 38% received 4 hours of therapy per day and 55% had an inpatient stay of more than 10 weeks (Table 2). SCI participants who did not have outpatient rehabilitation presented with 0.52 more EE than those who received outpatient therapy (*p* = 0.002).

**TABLE 2:** Duration since spinal cord injury and extent of treatment after spinal cord injury.

Patient detail	Factor	*n*	%
Duration since SCI	< 6 months	17	38
	≥ 6 months – 1 year	10	22.2
	> 1–2 years	6	13.3
	> 2 years – 5 years	5	11
	> 5years – 10 years	6	13.3
	> 10 years	1	2.2
Inpatient and outpatient therapy	No therapy after discharge as inpatient	3	6.7
	Outpatient therapy only at time of data collection	23	51.1
	Still in inpatient rehabilitation	19	42.2
Therapy hours per day	1 hour per day	1	2
	2 hours per day	4	9
	3 hours per day	13	29
	4 hours per day	17	38
	> 4 hours per day	10	22
Duration of rehabilitation hospital stay	< 2 weeks	0	0
	2–4 weeks	8	18
	> 4–6 weeks	4	9
	> 6–8 weeks	5	11
	> 8–10 weeks	3	7
	> 10 weeks	25	55

*n* = 45.

SCI, spinal cord injury.

Results pertaining to EE, distance, velocity and pulse before and after performing the 6MWT are shown in Table 3. Energy expenditure for the able-bodied participants was higher than for SCI participants (*p* < 0.001); however, EE/m was much higher for SCI participants than for able-bodied participants (*p* < 0.001).

**TABLE 3:** Energy expenditure.

Variables	SCI mean (± s.d.)	Able-bodied mean (± s.d.)	Mean difference between SCI and able-bodied	*p* value	95% confidence interval for difference between groups
EE (cal)	19.6	30.8	11.2	< 0.001	−16.50; −5.96
	10.1	9.8			
EE/m	0.33	0.08	0.25	< 0.001	0.12; 0.37
	0.29	0.02			
Distance (m)	114.1	388.7	274.6	< 0.001	−324.48; −224.84
	104.4	67.2			
Velocity (m/s)	0.32	1.1	0.76	< 0.001	−0.90; −0.63
	0.29	0.19			
Pulse before walking (bpm)	76.6	77.9	1.3	0.71	−7.88; 5.41
	13.6	9.9			
Pulse after walking (bpm)	106.4	87.7	18.7	< 0.001	10.29; 27.27
	17.7	11.8			

SCI, *n* = 45; Able-bodied, *n* = 21.

SCI, spinal cord injury; SD, standard deviation; EE, energy expenditure; cal, calorie; EE/m, energy expenditure per metre; m, metres; m/s, metres per second; bpm, beats per minute.

Mean EE/m was higher for the SCI participants older than 50 years (0.40 cal/m) and lower for those younger than 50 years (0.30 cal/m). Men had higher mean EE than women in both groups: men with SCI used 0.34 cal/m and able-bodied men 0.08 cal/m compared to 0.27 cal/m for women with SCI and 0.07 cal/m for able-bodied women. However, walking distance was greater for men in both groups (SCI participants: 121.9 m for men and 82.7 m for women; able-bodied participants: 395 m for men and 368.5 m for women). The EE/m increased with an increase in BMI for both SCI and able-bodied participants (SCI participants: 0.15 cal/m at a BMI of 16 kg/m^2^ – 16.9kg/m^2^, reaching 0.39 cal/m at a BMI of 30 kg/m^2^ – 35 kg/m^2^; able-bodied participants: 0.04 cal/m at a BMI of 17 kg/m^2^ – 18.49 kg/m^2^, reaching 0.11 cal/m at a BMI of 30 kg/m^2^ – 35 kg/m^2^). Mean EE/m was highest for ASIA A (0.71 cal/m) and decreased towards ASIA D (C: 0.29 cal/m; D: 0.26 cal/m) – with the exception of ASIA B (*n* = 1): 0.13 cal/m.

The mean EE was the highest for SCI participants walking with a walking frame and an orthotic (0.54 cal/m), followed by walking frame only (0.52 cal/m) and crutches only (0.18 cal/m). It was the lowest for SCI participants walking with crutch and orthotic or without a crutch or with orthotic only (0.16 cal/m for all these categories). Energy expenditure per metre of walking with crutches compared to a walking frame was significantly different (*p* = 0.03) and EE was 0.34 cal/m less for walking with crutches.

Results of the association between age, gender, BMI, velocity, pulse, ASIA classification, spasticity, length of rehabilitation stay, ROM and EE/m walked are shown in Table 4. There was a statistically significant association between an increase in walking velocity, hip flexion, hip extension and knee flexion ROM and decrease in the EE/m of walking.

**TABLE 4:** The correlation of age, gender, body mass index, velocity, pulse, American Spinal Injury Association (motor and sensory scores), spasticity, length of rehabilitation stay, range of movement with energy expenditure per metre respectively.

Variable	Correlation coefficient	*p* value
Age	0.20	0.30
Gender – male†	−0.10	0.51
BMI	0.01	0.97
Velocity	−0.60	< 0.001
Pulse after	0.20	0.13
Motor score (ASIA)	−0.30	0.08
Sensory score (ASIA)	−0.30	0.02
Spasticity – MAS for hip flexion	−0.02	0.91
Spasticity – MAS for hip extension	−0.02	0.87
Length of rehab stay	−0.10	0.56
Hip flexion ROM	−0.55	< 0.001
Hip extension ROM	−0.30	0.04
Knee flexion ROM	−0.30	0.03
Dorsiflexion ROM	−0.01	0.93
Plantarflexion ROM	0.20	0.23

BMI, body mass index; ASIA, American Spinal Injury Association; MAS, Modified Ashworth Scale; ROM, range of movement.

† Point biserial correlation.

Table 5 presents the multivariate stepwise regression analysis results for factors which had an influence on EE during walking. An inverse relationship was identified between EE/m walked and hip flexion ROM; for every degree decrease in hip flexion ROM there was a 0.003 increase in EE/m walked (*p* = 0.01). A unit decrease in velocity resulted in an increase of 0.41 in EE/m walked. ASIA A was used as the reference point in this analysis between ASIA classification and EE/m whilst walking. SCI participants with ASIA B, C and D had significantly less EE than those with ASIA A (*p* values are 0.05, 0.01 and 0.01 respectively).

**TABLE 5:** Multivariable analysis results for factors which had an influence on energy expenditure during walking.

Variable	Regression coefficient†	Standard error	*t*-test	*p* value	95% confidence interval
ROM: Hip flexion	−0.003	0.002	−1.67	0.01	−0.006 : −0.001
Velocity (m/s)	−0.41	0.15	−2.80	0.01	−0.70 : −0.11
**ASIA scores:**	-	-	-	-	-
ASIA B	−0.55	0.27	−2.07	0.05	−1.10 : −0.01
ASIA C	−0.38	0.13	−2.87	0.01	−0.64 : −0.11
ASIA D	−0.35	0.13	−2.69	0.01	−0.61 : −0.09

Note: Reference for ASIA scores is ASIA A classification.

ROM, range of movement; ASIA, American Spinal Injury Association.

† Effect with reference to baseline.

## Discussion

The mean age of the SCI participants was 38.9 years, which is generally younger than those in international studies (Kennedy *et al*. 2003; McKinley *et al*. 2007; Scivoletto *et al*. 2003). In a study by Joseph *et al*. (2015) on SCI which was conducted in Cape Town, the mean age of the participants was 33.5 years. The low average range between the current study and Joseph’s study in relation to other international studies may be as a result of the general lower life expectancy in South Africa compared to developed countries (World Bank 2009). Despite a worldwide increase in life expectancy for people living with SCI on the basis of technological and medical advances (Scivoletto *et al*. 2003) there are no published statistics on the life expectancy for such individuals in South Africa (Mothabeng 2011).

There were more male SCI participants (80%) than female in this study. This is in line with a reported male to female ratio of 4:1 (Wyndaele & Wyndaele 2006). Based on current literature, the male to female ratio of the incidence of SCI was found to be higher in less developed countries than in developed countries, probably because of the predominantly manual labour and greater risk taking behaviour of men in these countries (Ackery, Tator & Krassioukov 2004; Draulans *et al*. 2011; Van den Berg *et al*. 2010).

The SCI participants had a mean BMI of 23.9 kg/m^2^, which is within normal limits (WHO 2000). Body mass index values greater than 22 kg/m^2^ are considered as high risk for obesity and obesity-related chronic diseases in the SCI population (Laughton *et al*. 2009). Obesity-related comorbidities may affect the endurance and mobility of people with SCI (Laughton *et al*. 2009).

Most of the SCI participants (58%) were classified as ASIA D, with associated high sensory and motor scores. These high motor and sensory scores are expected to result in better gait efficiency because of the relationship between muscle strength and ambulatory capacity in SCI (Kim, Eng & Whittaker 2004). Thus patients with ASIA D SCI have to be considered for gait re-education.

A large proportion (46.7%) of the SCI participants presented with lesions above T10. The EE when walking is expected to be higher for this group. Kawashima *et al*. (2005) found that higher-level lesions resulted in higher EE in order to ambulate. Slower gait speed as a result of higher lesions result in excess load on the upper limb, making the gait less efficient (Kawashima *et al*. 2005).

Most of the SCI participants (38%) reported less than 6 months since SCI. Motor recovery has been reported to take place mainly during the first 6 months after injury (Kirshblum *et al*. 2004). However, walking ability improvements have been reported for individuals with lesions more than 3 years after SCI (Harkema *et al*. 2012). This would suggest that poorer mobility may be expected in this study group as a result of the limited recovery time, as 60% of participants reported less than a year since SCI.

Some SCI participants (6.7%) had no outpatient therapy after their initial inpatient rehabilitation and thus may not have reached their maximum functional capacity. Neurological recovery, including functional improvement in motor scores, and walking ability as a result of ongoing therapeutic intervention has been reported during the first and second year after injury (Burns *et al*. 2012). This would imply that once inpatient therapy has been concluded further recovery is still possible, which could be impeded by a lack of outpatient rehabilitation for the 6.7% of SCI participants in this study. In ideal circumstances the length of stay is determined by degree of motor recovery and potential for ambulation as well as patient goals. The majority of patients with SCI are discharged from hospital without having reached functional independence (Hastings, Ntsiea & Olorunju 2015). This may be ascribed to limited funding and resources (availability of beds in rehabilitation units), which is largely the determining factor of length of stay (Mothabeng 2011).

Mean EE/m of SCI participants was four times higher than that of able-bodied participants (*p* < 0.001) (Table 3). This is lower than the results reported by Kawashima *et al*. (2006), who found EE to be approximately six times higher for SCI participants who walk with orthotic-assisted gait than for able-bodied participants. However, in Kawashima’s study, the SCI participants used the reciprocating gait orthotics and had complete paraplegia between T5 and T12, which may have contributed to higher EE. In this study, incomplete SCI lesions were included and a variety of assistive devices used to aid walking. The higher EE for SCI participants compared to able-bodied individuals can be attributed to greater upper limb and trunk involvement during walking in order to swing their paralysed lower limbs and maintain balance (Kawashima *et al*. 2006). Thus it is not surprising that ambulatory EE is significantly greater for SCI participants than for able-bodied participants. This indicates the need for provision of appropriate assistive devices to improve walking pattern and make walking energy efficient for people with SCI.

The EE for the able-bodied individuals was higher when distance was not taken into consideration. It is reported that walking velocity in people with SCI is slower than in the able-bodied population (Ditunno *et al*. 2007). The current study has shown that with a unit decrease in velocity the EE/m increases by 0.41 (Table 5). The mean distance covered in the 6MWT for the SCI sample in this study was 114.10 m (SD ± 104.40) compared to 338.70 m (SD ± 67.20) for the able-bodied sample (*p* < 0.001). Thus the able-bodied individuals would have expended more energy than the SCI individuals to cover this distance. Energy expenditure per metre was much higher for the SCI participants than for able-bodied participants, indicating that the distance resulted in more EE for able-bodied individuals.

Energy expenditure when walking with crutches was 0.34 cal/m less than when using a walking frame (*p* = 0.03). These findings are similar to the results reported by Ulkar *et al*. (2003), who also found crutches to be more energy efficient than walkers. However, in that study, the participants walked with both assistive devices in turn to control for differences in the two groups. Walking with a walking frame and an orthotic yielded the highest EE value of all the assistive devices in this study, with a mean of 0.54 cal/m. The able-bodied participants yielded EE/m of 0.08 cal/m, thus the walking frame and orthotic group required 6.75 times the energy of the able-bodied group. Similar results were found by Kawashima *et al*. (2006), who reported that orthotic-assisted SCI gait required six times the energy cost during walking compared to able-bodied gait. The high EE cost of walking frame ambulation could be ascribed to slow walking speed, step-to-gait pattern and the repeated lifting of the frame necessary for forward motion (Priebe & Kram 2011).

In the correlation analysis, overall inverse relationship was observed between EE/m and the following: hip flexion, hip extension, knee flexion ROM and velocity. Kawashima *et al*. (2006) also established that hip ROM affects the EE whilst walking. Higher thoracic lesions and resultant increased paralysis in the trunk and hip musculature produce smaller ROM at the hips during walking. Insufficient leg swing is therefore produced, which leads to slower gait speed, shorter distances and higher EE. Ability to flex the knee has also been found to contribute to a reduction in energy consumption during walking (Baardman *et al*. 2002). This indicates the need to maintain or improve knee flexion ROM during rehabilitation of patients with SCI.

Velocity and EE during walking yielded a significant inverse relationship. Walking velocity in people with SCI is slower than in the able-bodied population and they often require assistive devices (Ditunno *et al*. 2007; Van Hedel & EMSCI Study Group 2009). SCI participants with an ASIA A classification walked at a mean velocity of 0.05 m/s and utilised 0.71 cal/m whilst walking. Participants with an ASIA D classification walked at 0.38 m/s and their EE was 0.26 cal/m, indicating that ASIA D participants walk faster but with less EE.

Energy expenditure per metre decreased from ASIA A to ASIA D. Participants with an ASIA B classification had lower EE/m than any of the other categories; however, this category consisted of one individual only, which could explain the skewed data. The differences in EE between different ASIA classifications can be explained by the fact that walking function regained after a SCI depends strongly on the level and completeness of the injury (Van Hedel & Dietz 2010).

## Conclusion

There are significant differences for EE whilst walking between the able-bodied and SCI populations. Factors to consider in order to maximise energy efficiency whilst walking are hip flexion ROM, walking velocity, and encouraging continued therapy beyond inpatient rehabilitation. Selection of a walking device that optimises EE whilst walking should be considered when clinically relevant.

### Limitations of the study

The walking aids and orthotics used during the assessment were those that the participant usually used for walking. However, there is quite a difference in resources between rehabilitation units. Where knee extension was absent, some participants had callipers to compensate, whereas some from poorly resourced rehabilitation units only had backslabs and bandages to secure to the participant leg. Furthermore, a lack of ankle dorsiflexion to achieve swing-through is compensated by an ankle foot orthotic (carbon or plastic), which was not available to some of the participants from poorly resourced rehabilitation units. This causes circumduction during gait, with potential balance, trunk and upper limb loading, which may increase EE.
